# Supplementary material for: Learning curve and surgical outcome of robotic assisted colorectal surgery with ERAS program
Source: Sci Rep. 2022 Nov 29;12:20566. doi: 10.1038/s41598-022-24665-w (PMC9709162; doi:10.1038/s41598-022-24665-w)
Supplement: Supplementary file 1 — Supplementary Information 1. [file 41598_2022_24665_MOESM1_ESM.zip › LSC Raw data-Ñ[▒K/096/O_ñw╝╨░O▒Kñσ.pdf]

正式摘要(電子簽章：000000200496986)

請選擇...reporter

臺中榮民總醫院

Operation Note

[redacted]

- 手術日期：2020/10/07
- 手術醫師：VS 蔣鋒帆，CR 林陳彥，Fellow 林昌霖，R 楊捷儒
- Anesthesia：GE
- Position：Supine
- Aseptic agents：Betadine Aqua，Zephnan

Diagnosis

- Pre-operative diagnosis : Malignant tumor of proximal T colon
- Post-operative diagnosis : Malignant tumor of proximal T colon s/p laparoscopic right hemi-colectomy

Surgical Indication

- For removing the primary tumor

Operation

- 1. Laparoscopic right hemicolectomy with side to side anastomosis (OpenGIA 80-3.8 X2 were applied)
  2. Ileo-colic artery, right colic artery and middle colic artery were all ligated via Hemo-clips.
  3. Take down of hepatic flexure : (+)
  4. Cut margin: proximal (ileum) 4 cm, (colon) >15 cm, distal 8 cm.
  5. Repair of mesentery defect : Nil.
  6. No drainage tube was placed.

Finding

- 1. A 3 X 4 cm tumor over proximal T-colon area. Suspicious enlarged lymph nodes at ileocolic artery area.
  2. No other gross tumor was found.
  3. There was no visible liver metastasis or visible peritoneal seeding.
  4. An ecchymosis patch was noted over distal T-colon area. No progression of the ecchymosis area.

Procedure

- 1. General endotracheal anesthesia was induced and then the patient was placed in the supine position.
  2. Preoperative antibiotics were given. A Foley catheter and nasogastric tube were placed. The abdomen was prepped and draped in standard sterile fashion.
  3. A small vertical incision was made left to the umbilicus and a 12mm trocar was inserted into the peritoneal cavity by open method.
  4. Pneumoperitoneum was established with CO2 inflation. Laparoscope was inserted and then a 12mm trocar were inserted over LUQ
  of abdomen.
  5. Another 5mm trocar was inserted over LLQ of abdomen ; The other 5mm trochar was inserted over RLQ of abdomen.
  6. The small bowel was inspected and retracted to downward by grasper. The peritoneum overlying the mesentery was then scored with electrocautery and the ileocolic artery was identified, ligated with endoclips, and transected via Harmonic scalpel.
  7. The remaining vessels to the cecum were also divided and ligated. Using electrocautery and Harmonic scalpel, omentum was freed from the transverse colon and lesser sac was entered.
  8. The middle colic artery and right colic artery were all further ligated via Hemo-clips and Harmonic scalpel.
  9. After the vessel were all under controlled, the colon was freed from its peritoneal attachments along the line of Toldt from the cecum to the hepatic flexure.
  10. Hemostasis was checked in the operative field. Cecum, ascending colon and hepatic flexure were mobilized and then laparoscope was removed.

- 11. The midline incision was extended over left peri-umbilical area.
  12. The ascending colon was delivered into the incision and points of transection were selected proximally and distally.
  13. Side to side anastomosis was performed with two OpenGIA 80-3.8mm. Interrupted 3-0 silk Lambert sutures were used to re-inforcement at outer layer
  14. The anastomosis was checked and found to be intact and widely patent.
  15. The small bowel and colon was put back into the abdomen. Pneumoperitoneum was established again.
  16. Possible bleeder was examed again. The orientation of the small bowel was checked thoroughly.
  17. The fascia was closed with interrupted sutures via Num.1 Vicryl. The fascia of the 12 mm trochar wound was closed with 1-0 Vicryl.
  18. The skin wound was sutured with interrupted method via 3-O Vicryl, then covered with Surgicel (Gel form) and Medipore.
  19. The patient tolerated the procedure well and was brought to the POR in stable condition.
- Estimated blood loss: minimum
- 器械數量: 正確
- 紗布數量: 正確

Image

- PAINT
  - abdomen

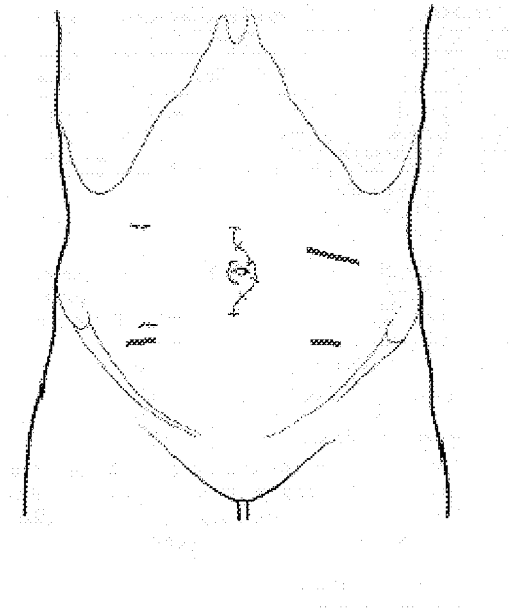

  - colon & rectum

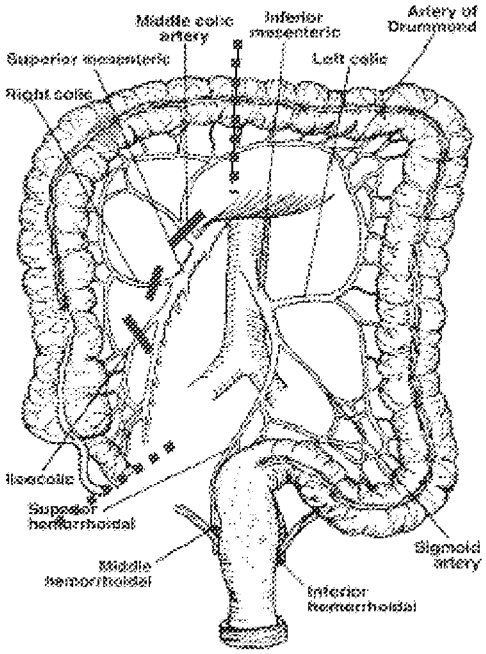

- The tumor could be seen clearly as pic below

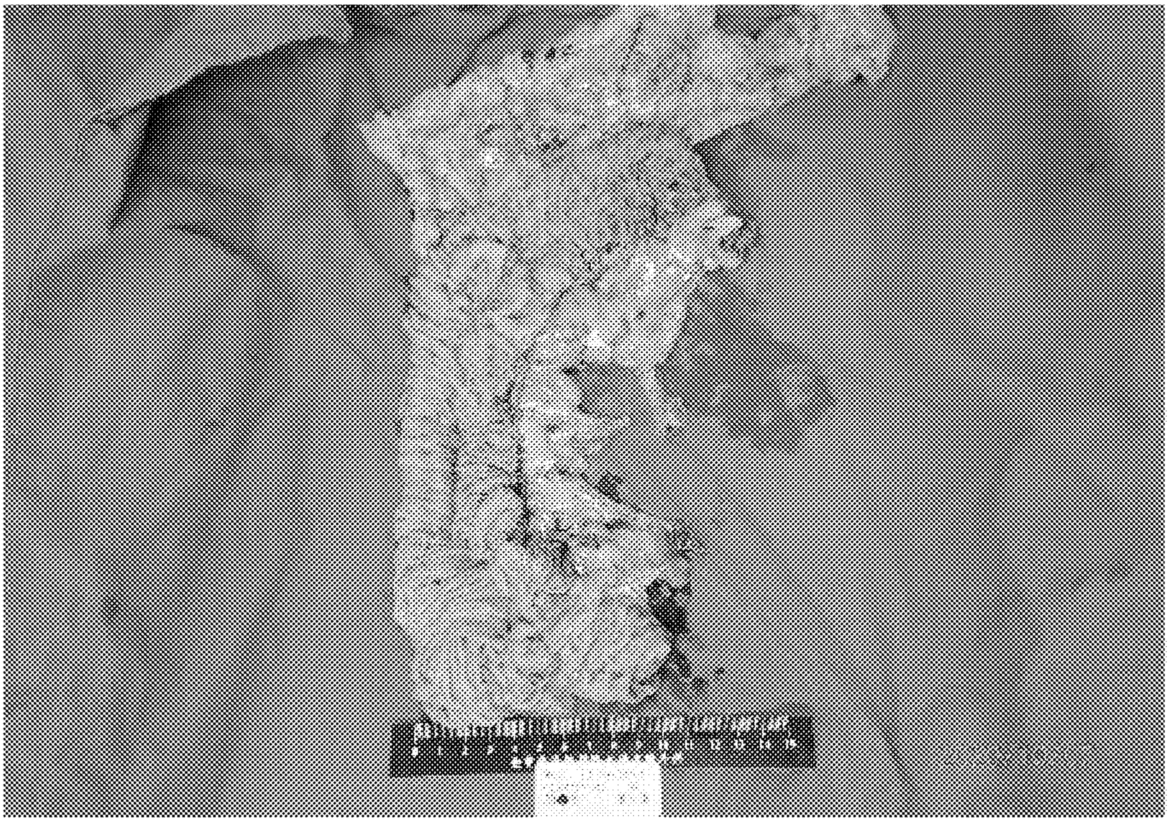

主任 2062F蔣鋒帆 / 醫師 2301H林陳彥

正式摘要(電子簽章：**000000200496986)**

請選擇...reporter

doctorRank:B
